# Public involvement in chronic respiratory diseases research: A qualitative study of patients', carers' and citizens' perspectives

**DOI:** 10.1111/hex.13917

**Published:** 2023-12-25

**Authors:** Margarida Areia, Liliana P. Dias, Paula Matos, Daniela Figueiredo, Ana L. Neves, Emília D. da Costa, Cláudia C. Loureiro, José L. Boechat, António B. Reis, Pedro Simões, Luís Taborda‐Barata, João A. Fonseca, Ana Sá‐Sousa, Cristina Jácome

**Affiliations:** ^1^ Allergy and Clinical Immunology Unit Centro Hospitalar Vila Nova de Gaia/Espinho Vila Nova de Gaia Portugal; ^2^ CINTESIS@RISE, Health Research Network, Faculty of Medicine University of Porto Porto Portugal; ^3^ CINTESIS@RISE, School of Health Sciences University of Aveiro Aveiro Portugal; ^4^ MEDCIDS–Department of Community Medicine, Information and Health Decision Sciences Faculty of Medicine, University of Porto Porto Portugal; ^5^ Department of Primary Care and Public Health Imperial College London London UK; ^6^ Design Department Faculty of Fine Arts, University of Porto Porto Portugal; ^7^ Pneumology Unit Hospitais da Universidade de Coimbra, Centro Hospitalar e Universitário de Coimbra Coimbra Portugal; ^8^ Department of Pathology Basic and Clinical Immunology, Faculty of Medicine of the University of Porto Porto Portugal; ^9^ Allergy Unit Instituto CUF Porto e Hospital CUF Porto Porto Portugal; ^10^ Utopia Academy, Utopia, Innovation on Digital Media Lab Universidad Carlos III de Madrid Madrid Spain; ^11^ CICS‐UBI Health Sciences Research Centre, CACB‐Clinical Academic Centre of Beiras University of Beira Interior Covilhã Portugal; ^12^ UBIAir—Clinical and Experimental Lung Centre, CACB‐Clinical Academic Centre of Beiras University of Beira Interior Covilhã Portugal; ^13^ UCSP Fundão, ACeS Cova da Beira Covilhã Portugal; ^14^ Department of Immunoallergology Covada Beira University Hospital Centre, CACB‐Clinical Academic Centre of Beiras Covilhã Portugal

**Keywords:** asthma, chronic obstructive pulmonary disease, chronic respiratory disease, citizen science, patient and public involvement

## Abstract

**Introduction:**

Patient and public involvement (PPI) initiatives involving patients with chronic respiratory disease (CRD) are rare. Therefore, this study aimed to explore the perspectives of patients with CRD, carers and interested citizens regarding the relevance and need for a PPI network and suggestions for its implementation.

**Methods:**

A qualitative study based on focus groups was conducted. Recruitment occurred through invitations on social media platforms and to patients who have participated in previous asthma studies of the team. Three focus groups were conducted, via video conference, using a semi‐structured guide. Thematic analysis was performed by two independent researchers and discussed with the extended team.

**Results:**

Fifteen patients with CRD, one carer and one interested citizen (13 females, median 36 (range: 18–72) years) participated. All participants acknowledged the importance of implementing a collaborative network and demonstrated interest in being integrated. Participants acknowledged the importance of their involvement in several phases of the research cycle. The main aim identified for this network was to facilitate communication between patients and researchers. Participants regarded the integration of patients, carers, researchers and healthcare professionals from different scientific areas as relevant. The use of digital platforms to attract members and support the work, together with group dynamics and regular meetings, were some of the most relevant practical considerations for implementing the network. The identified facilitators for their engagement were sharing experiences, researchers' and healthcare professionals' support and feedback and schedule flexibility. The identified barriers included the amount of time dedicated, low health/digital literacy and the potential detachment of nondiagnosed patients or those with low symptom impact in daily life.

**Conclusion:**

Patients, carers and citizens acknowledged the relevance of implementing a collaborative network and demonstrated interest in active participation in every stage of the health research cycle. A deeper knowledge of the barriers and facilitators identified in this study could support implementing these initiatives in Portugal.

**Patient or Public Contribution:**

This study was designed by a research team that included one patient with asthma and one carer. They were specifically involved in building the study protocol and the interview guide. They also gave feedback regarding the electronic consent form and the short sociodemographic questionnaire created, namely by removing noncontributing words or phrases and rewording expressions. The lay summary was written by another patient with asthma. All participants of this study were invited to implement and integrate the ConectAR network—a collaborative network of research in respiratory health.

**Public Summary:**

In Portugal, chronic respiratory patients do not have an active role as ‘coinvestigators’. This study aimed to acknowledge if patients and citizens considered a patient and public involvement network useful, whose main purpose would be to facilitate communication between patients and researchers. A study based on online group interviews was carried out with patients with chronic respiratory diseases and interested citizens, both recruited on social media platforms. Participants considered that bringing together patients, carers, researchers and healthcare professionals is valuable because sharing different experiences and perspectives may help patients to improve their daily lives and increase research quality. In conclusion, patients agree that implementing a collaborative network with researchers and healthcare professionals and participating in the health research cycle is quite preponderant. Acknowledging what can help and deter this network may be beneficial to implementing this type of initiative in Portugal.

## 1 BACKGROUND

Asthma affects 350 million people worldwide and chronic obstructive pulmonary disease (COPD) around 200 million, with the latter being the third‐leading cause of death worldwide.^1^ Asthma and COPD are two of the most common chronic respiratory diseases (CRD) worldwide, being a source of substantial burden with a high personal and social impact.^2^

Management of CRD is a challenge, globally. Several studies have reported that poor understanding of the diagnosis, nonadherence to treatment, or its inadequate use are still major barriers related to the management of patients with CRD, leading to increased healthcare costs and poor clinical outcomes such as exacerbations, hospitalisations, decrease in quality of life and early mortality.^3^, ^4^, ^5^ There is a growing need for multidisciplinary management of patients with CRD, characterised by integrative strategies and the involvement of various healthcare professionals, patients and carers, from diagnosis to treatment.^6^, ^7^ Additionally, patients diagnosed with CRD expect disease management practices to consider their circumstances, motivations, expectations and beliefs.^8^ To address patients' multidimensional needs and improve health outcomes, healthcare systems need to be centred on the patient when delivering care and researching innovative approaches.

Patient and public involvement (PPI) is gaining recognition as a key component of patient‐centred care.^8^ PPI in research refers to ‘research being carried out ‘with’ or ‘by’ members of the public (including patients, potential patients, carers and people who use health and social care services) rather than ‘to’, ‘about’ or ‘for’ them’.^9^ Patients contribute with a different and complementary perspective to research, by for example adding personal knowledge and lived experience, bringing new research ideas and ensuring research priorities are aligned with their needs. PPI in health research is thus believed to improve its research quality and relevance and is increasingly an ethical imperative.^10^

Implementation of PPI in research is imperative, despite posing challenges in terms of time expenditure, required training and support, and power sharing.^11^ Organisations such as the National Institute for Health and Care Research (NIHR) and Canada's Strategy for Patient‐Oriented Research are examples of the successful establishment of PPI in health research.^9^, ^12^ In the respiratory field, the European Lung Foundation has been remarkable in the involvement of patients with CRD and other lung conditions in research and guideline development.^13^ In Portugal, as in other countries, however, there is no established network involving patients with CRD as ‘coinvestigators’.^14^, ^15^

Different frameworks for supporting and evaluating PPI in research exist, but codesign of adapted frameworks to meet specific/local needs and contexts is advised. The first step to guide the development of the framework was to listen to patients, carers and citizens to obtain their views regarding involvement in research and a PPI network. Some key principles in engaging patients with CRD as study partners are known, such as clarifying the nature of involvement; integrating patients' disease‐related knowledge and perspective; and promoting patient‐to‐patient dissemination.^8^ These principles may also apply to the Portuguese context, but cultural differences may also exist and have to be resolved.^8^, ^16^

Therefore, this study aimed to explore the perspectives of Portuguese patients with CRD, carers and interested citizens regarding the relevance and need for a PPI network and suggestions for its implementation.

## 2 METHODS

### 2.1 Study design

An exploratory qualitative study using focus groups was conducted with patients with CRD, carers and interested citizens. This work is part of a larger protocol that aims to develop a network to promote the involvement of patients and the public in health research.^17^ Focus groups were the approach chosen to allow interaction, sharing of experiences and capturing data on individual needs and beliefs.^18^ This study was reported following the Consolidated Criteria for Reporting Qualitative Research checklist.^19^ A convenience, nonprobabilistic sampling method was used. Participants were recruited using social media platforms and through direct invitation to patients who participated in previous epidemiological or observational asthma studies of the patient‐centred innovation and technologies research team. Invitations included an online form informing about the study and allowing persons to express their willingness to participate. Then a researcher contacted the interested participants to confirm their willingness to engage in the study, check eligibility criteria and obtain electronic informed consent.

### 2.2 Participants

Participants were eligible if they were (a) patients with CRD, carers, or interested citizens; (b) 18 years old or older; (c) able to understand the purpose and procedures of the study and (d) able to communicate and express opinions in Portuguese. Exclusion criteria included the inability to participate in the scheduled focus group or to provide consent.

### 2.3 Data collection

Three focus groups were conducted between March and April 2022. Before the focus group, participants completed a questionnaire about sociodemographic data (e.g., age, gender, current occupation). Each focus group was composed of 4–8 participants. On average, focus groups lasted 74 min (min–max: 63–82). Due to the COVID‐19 pandemic, focus group sessions were conducted via video conference using the online Zoom platform^20^ and moderated using a semi‐structured guide (Supporting Information S1: Appendix 1). Two experienced moderators conducted the focus group, C. J. conducted two and A. L. N. one. C. J. is a female physiotherapist who holds a PhD and has extensive knowledge of comprehensive interventions for CRD and qualitative studies. A. L. N. is a general practitioner and researcher with experience in qualitative methodologies. Both have experience with PPI in research. Two observers (A. S. S. and M. A.) were present in each focus group for logistic support and to take observational notes of the group dynamics and main subjects of discussion. Focus groups were audio‐recorded and transcribed verbatim.

### 2.4 Data analysis

Thematic analysis was used to analyse the interview transcripts.^21^ The interview guide, based on the work of Poureslami et al.,^8^ provided a framework for exploring specific aspects of participants' views and therefore data was coded to fit into two comprehensive topics: (i) willingness to participate in a PPI initiative; and (ii) suggestions to implement PPI. After this, the data were ‘open‐coded’ to best represent meaning as communicated by participants. This semi‐inductive approach was adopted to emphasise respondent/data‐based meaning; nevertheless, a degree of deductive analysis was applied to ensure that the open coding contributed to producing themes that were relevant to the study goals.^22^ Braun and Clarke's^21^ recommendations for data categorisation were followed, explicitly: (i) familiarisation with the data (the interviews were transcribed, then the transcripts were read and reread and initial ideas were noted down for a comprehensive understanding of all aspects of the data); (ii) generation of initial codes (codes were initially identified by two independent researchers); (iii) searching for themes (relevant data extracts were combined or allocated according to the overarching themes); (iv) reviewing themes (themes were reviewed to allow for clearer identification and distinction between them); (v) defining and naming the themes (names of themes within the data were identified to capture the essence of each theme concisely); and (iv) produce the report (the most demonstrative quotes were selected to exemplify the themes).^21^

To ensure the credibility, trustworthiness and rigour of the data, the recommendations of Nowel et al.^23^ were considered, namely, researcher triangulation, reflexivity and member checking. Therefore, data were initially analysed by two independent researchers (M. A. and L. D.). The two researchers attended periodic meetings with other research team members (C. J., A. S. S., A. L. N., D. F.) to critically discuss the analysis process and reflect on how their pre‐existing knowledge, biases and personal experiences could affect it. Credibility was also guaranteed through the process of member checking, as the proposed data summary and relevant quotations were reviewed and commented on by one patient who participated in the focus groups.

### 2.5 Ethical considerations

The study was conducted in accordance with the Declaration of Helsinki and received approval from the Ethics Committee of the Faculty of Medicine of the University of Porto (28/CEFMUP/2021). Electronic informed consent from all participants was obtained before the focus group meetings.

## 3 RESULTS

### 3.1 Participants

Twenty‐two participants were willing to participate, but 17 effectively attended. The most common reason for nonparticipation was a conflict with the focus group schedule. All participants were Caucasian, had a median of 36 years and most were female (*n* = 13, 76%). Participants mainly were patients (*n* = 15, 88%), 14 with asthma and one with COPD. Most participants were active workers (*n* = 11; 65%) and from the Littoral North of Portugal (*n* = 12; 71%). (Tables 1 and 2).

**Table 1:** Participants' characteristics (*n* = 17).

Characteristics	
Female	13 (76%)
Age, median (range)	36 (18–72)
Role	
Patient	15 (88%)
Carer	1 (6%)
Citizen	1 (6%)
Occupation	
Active worker	11 (65%)
Student	4 (23%)
Retired	2 (12%)
Country region	
Littoral North	12 (71%)
Centre	3 (17%)
Lisbon metropolitan area	2 (12%)

*Note*: Values are shown as *n* (%) unless otherwise indicated.

**Table 2:** Summary of the generated themes for each of the two topics of interest according to participants' perspectives.

*Topic 1*	*Willingness to participate in a PPI initiative*
Level of engagement	‘I think this is where the patient comes in, because a researcher or clinician who doesn't have a disease, doesn't have the same issues that the patient has’. (Patient with asthma, 30 years old) ‘The communication between everyone could lead to the identification of questions, needs, more suited to people's daily lives’. (Patient with asthma, 30 years old) *‘*Scientists can have an opaque language, if you need to change that to simple language, prepare posters, make PowerPoints or things like that to disseminate information, you can count on me’. (Patient with asthma, 45 years old)
Previous experience	‘It was great because now I have better control of my asthma’. (Patient with asthma, 18 years old) ‘By entering this project, I felt much more included … a group, where we could talk about our problems … for a person not to feel alone’. (Patient with asthma, 24 years old)
Relevance	‘It is very important to participate in the projects so that we can increase our knowledge and we can communicate and say better how we are feeling’. (Patient with asthma, 54 years old) *‘*We won't feel alone’. (Patient with asthma, 18 years old) ‘In terms of how we feel, the treatments and the network may help too. If you are aware of other people who suffer from the same disease as you, I think this helps to better handle the situation, which is not easy’. (Patient with asthma, 54 years old) ‘I would regard as more important researchers being aware of how we feel because projects are often made for patients, but patients do not give an opinion and researchers do not know what is going on’. (Patient with asthma, 54 years old) ‘Because research should answer the questions, not only of the researcher and the healthcare professional but also of the patient, to have a greater impact on quality of life’. (Patient with asthma, 30 years old) ‘I think that science evolves through the investigations of those who investigate and through our help, so it will be possible to achieve solutions that are a little more practical and more effective, and only through our experience will you be able to change these things, only you can do it’. (Patient with asthma, 39 years old)
Personal motivation	‘People can see that that group is useful for something and that it is useful, and to see that their day‐to‐day life is somehow different because they have that form of communication’. (Patient with asthma, 30 years old) ‘The motivation, in this case, to help other patients, to make a contribution to science’. (Patient with asthma, 33 years old) ‘For example, access to something that could be related to asthma or not, a voucher, or a pulmonology consultation, an allergy consultation, or a physiotherapy consultation’. (Patient with asthma, 38 years old) ‘When participants are rewarded in some monetary way there is a greater responsibility and they always try to do better’. (Patient with asthma, 36 years old)
*Topic 2*	*Suggestions to implement PPI*
Aims	‘I think that the main objective should be the communication between doctor and patient, between researcher and patient…’. (Patient with asthma, 18 years old) ‘The language … we as patients passing on what was said to other patient, facilitates his understanding’. (Patient with asthma, 54 years old)
Stakeholders involved	‘I think the connection between nurses, carers, doctors is important’. (Patient with asthma, 54 years old) ‘I think there should be much more people involved and they should work as a multidisciplinary team. Patients need all areas’. (Citizen, 36 years old)
Communication strategies	‘In social networks we have people with high and low socioeconomic incomes, older people’. (Patient with asthma, 23 years old) ‘I think the platform to be used to keep people engaged will be important and maybe it's one of the most important things, maybe even more than the content itself’. (Patient with asthma 30 years old) ‘When it is proposed by someone they trust, the pulmonologist or the allergist, who says “take part in this project because I think it is an important project for your disease, because will help you—I think they are more susceptible to wanting to join”’. (Patient with asthma, 23 years old)
Network workflow	*‘*I think it would be good to have regular, pre‐scheduled meetings so that everyone is available on a certain date. These meetings could be held online’. (Patient with COPD, 72 years old)
Facilitators	‘It is pertinent that researchers are in this group. I think it is very important to give credibility, to accompany, to give power to the words of asthmatics’. (Carer, 57 years old) ‘For people to talk, you need to build trust between people’. (Carer, 57 years old)
Barriers	*‘*The lack of time that people have to make themselves available for such an embryonic project’. (Patient with asthma, 45 years old) *‘*Older people who may not use digital platforms can be a difficulty, and then we end up having only a younger group of people with easier access to technology’. (Patient with asthma, 30 years old) ‘One of the difficulties may be to attract people with a less serious illness who may not see a real need for this network to exist’. (Patient with asthma, 30 years old) *‘*The participation will be conditioned by my restrictions due to the disease and these restrictions will always have to be analysed case by case, project by project…’. (Patient with COPD, 72 years old)

Abbreviation: PPI, patient and public involvement.

### 3.2 Qualitative findings

**Figure 1:**
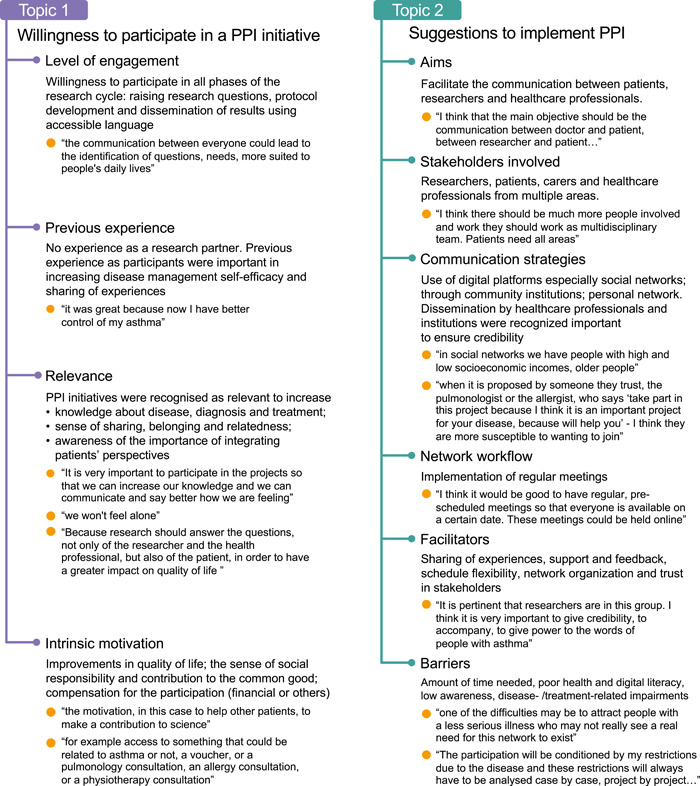
Thematic map presenting the two topics and generated themes according to participants' perspectives.

Parallel to the two aims of this work, two topics were explored within the thematic analysis: Topic 1—Willingness to participate in a PPI initiative, where four themes were identified; and Topic 2—Suggestions to implement PPI, with its six themes (Figure 1).

#### 3.2.1 Topic 1: Willingness to participate in a PPI initiative

Theme 1 (Level of engagement) Participants expressed their willingness to participate in all phases of the research cycle. They mentioned their potential collaboration in raising research questions, protocol development, including the validation of questionnaires and results' dissemination in the scientific community and society. Participants reinforced the need to use accessible language in scientific outputs.

Theme 2 (Previous experience) None of the participants had previous experience as a research partner. Five had collaborated as participants in research projects about digital applications to promote medication adherence and respiratory rehabilitation. These patients acknowledged the importance of their previous experiences in increasing disease management self‐efficacy and promoting the sharing of experiences among other patients.

Theme 3 (Relevance) Participants recognised the relevance of the implementation of PPI initiatives. Participants expressed their willingness to share their knowledge and experience of the disease, as they recognised patients/carers as experts in their own illness experiences/conditions. Participants also voiced their desire to increase their knowledge about their disease, diagnosis methods and treatment options. Some patients mentioned the disease burden affecting social and working life and the need for support, considering that the implementation of this kind of network could increase their sense of sharing, belonging and relatedness.

Participants voiced the need to increase awareness of the importance of integrating patients' perspectives among healthcare professionals and researchers. Some participants also mentioned the importance of having feedback on their contribution and stressed the importance of valuing patients as co‐researchers. According to them, a patient‐centred research model would impact the research outcome by increasing its quality and ensuring that the results meet the patients' real needs.

Theme 4 (Personal motivation) Expectations of improvements in quality of life due to public involvement in the research were emphasised as the main motivation to participate in such a network. Participants also expressed a sense of social responsibility and the willingness to contribute to the common good. Participants also stated that reciprocity could be useful for the maintenance of patients, carers and citizens in the network, for example through the use of vouchers for medical appointments, physiotherapy and physical activities.

#### 3.2.2 Topic 2: Suggestions to implement PPI

Theme 1 (Aims) According to participants, this network should aim to facilitate communication between patients, researchers and healthcare professionals by creating a communication channel and promoting a patient‐friendly language.

Theme 2 (Stakeholders involved) Participants suggested that the network should include researchers, patients, carers and healthcare professionals from different areas: physicians, psychologists, physiotherapists, nurses and speech therapists.

Theme 3 (Communication strategies) Participants proposed using digital platforms, especially social networks, for recruitment and network amplification. To ensure that the network can represent the full spectrum of patients/carers, including underrepresented groups (e.g., older people, people with low education level/deprived backgrounds), participants suggested the dissemination of the network in the community, for example, schools, churches, social groups and city halls. Participants also voiced their availability to collaborate by spreading the network among family and friends. Participants mentioned the dissemination by healthcare professionals and institutions as an important communication strategy and a way to ensure credibility to the network.

Theme 4 (Network workflow) Participants voiced the importance of creating group dynamics and suggested implementing regular meetings, both in‐person and online, to define aims and work methodology.

Theme 5 (Facilitators) Participants identified as facilitators for public engagement: sharing of experiences, researchers and healthcare professionals' support and feedback, schedule flexibility, network organisation and trust in stakeholders and the initiative.

Theme 6 (Barriers) Regarding public involvement constraints, participants identified the amount of time dedicated as the main factor influencing engagement. Poor health and digital literacy, and low awareness of those underdiagnosed or with mild symptoms were also identified as main barriers to engaging in the network. Disease and treatment burden were also expressed by two participants as potential barriers

## 4 DISCUSSION

This qualitative research generated knowledge about patient and public willingness to participate in research, and practical considerations for the implementation of a network with such aim. Overall, patients, carers and citizens acknowledged the relevance of the implementation of a collaborative network and demonstrated interest in active participation in every stage of the health research cycle. They also voiced the main facilitators and barriers to the implementation of such an initiative.

PPI should be integrated into research projects at the earliest opportunity and not only after decisions have been made, allowing maximal contribution.^24^ Participants' perspectives are aligned with NIHR, an organisation that supports appropriate PPI in research and considers that patients can be involved in every stage of the research cycle: identifying and prioritising, commissioning, designing, managing, undertaking, disseminating, implementing and evaluating impact.^9^ This involvement may happen in several forms: consultation (patients give their opinion on specific issues), collaboration (shared decisions between researchers and patients) and patient‐led research (patients deliver and manage research).^25^, ^26^ From the participants' views, it can be seen that they were more keen on an active approach of PPI, related to collaboration/patient‐led research rather than consultation. This was somewhat expected, as active approaches to PPI are known to be more encouraging and are more frequently reported in the literature.^16^ One of the examples provided was the support of patients in the development of questionnaires. Indeed, a review shows that patient involvement in the development of Patient‐Reported Outcome Measures is important to ensure that health measures are relevant and patient‐centred, yet patients are rarely involved and few studies involve patients in the early phases.^27^

Participants' expectations were mainly related to increasing their health literacy, sense of belonging and the positive impact on the research, which have been previously described in other PPI initiatives.^28^, ^29^ Nevertheless, we need to consider that these expectations are voiced by participants without previous experience as research partners. Thus, it is advisable to have an ongoing assessment of expectations as network members gain research experience. Management of expectations is one of the tips for successful patient‐involving partnerships.^30^

Participants perceived the network as an opportunity to facilitate communication between patients, researchers and healthcare professionals. The communication expectations are not specific to our context, as they have also been previously highlighted by patients with CRD from Canada^8^ but also by patients/carers with other health conditions.^31^, ^32^, ^33^ Indeed, clear and effective communication is one of the main focus of partnership‐focused PPI initiatives.^10^ Different studies have identified that clear information and communication are associated with greater involvement in research, as well as with better disease management.^34^, ^35^, ^36^ This also links with the participants' advice to always use accessible and lay language among stakeholders and in all communication strategies (digital platforms, contacts with the community/healthcare institutions).^8^, ^37^

The identified facilitators for the patients/carers/citizens' engagement were the sharing of experiences, researchers' and healthcare professionals' support and feedback, confidence in the project and schedule flexibility. Some of these tips were also raised in previous PPI initiatives, namely flexible working arrangements.^10^, ^30^ Regarding practical considerations, the organisation of regular activities to maintain contact and reimbursement measures are aligned with known frameworks.^10^, ^30^, ^38^

The desire to know the results of their contribution was expressed by our participants. Both researchers and healthcare professionals need to demonstrate input is valued, as suggested by Turner et al.^30^ Nevertheless, reporting outcomes of public involvement may be challenging because PPI is not objective or easily quantified and its wider impact could be missed.^39^, ^40^ Previous PPI initiatives suggest the involvement of patients and the public on committees, grant applications and publications as possible key outcomes.^30^ Current PPI initiatives can start measuring their impact by using these outcomes, but there is space to work with patients and come up with novel approaches in future.

Patient involvement was considered overall beneficial for health research, but barriers were also identified. In our study, the amount of time dedicated was the main factor influencing patients' engagement. Other identified barriers included low health/digital literacy and the potential detachment of underdiagnosed patients or those with low symptom impact on daily life. Several studies have also identified different attributes that help to explain involvement in PPI and other initiatives, including age, gender, education, time to be involved, accessibility and timing of meetings and health condition.^34^, ^41^, ^42^ Nevertheless, underdiagnosis or severity of disease were newly reported factors and may inform future dissemination strategies of the network. A future PPI network should be accessible to people from underrepresented groups like deprived backgrounds and those who are not healthy or digitally literate.

This work has some limitations that need to be acknowledged. It is based on a convenience sample recruited through digital media (social networks, e‐mail). This may have introduced a selection bias, favouring the participation of younger patients with better digital literacy. Inviting people who have previously participated in asthma studies could have introduced social desirability bias, as these participants could be more likely to report positive feedback and desire to participate. This also introduced a bias for the inclusion mainly of patients with asthma. Moreover, only one carer and one interested citizen participated, demonstrating the researchers' difficulty in attracting this public and integrating their vision. Challenges in recruiting these stakeholders have been previously acknowledged.^43^ Despite the selection bias, this work was a necessary first step to give voice to patients and the public and start the process of cocreating a PPI network in respiratory research. Future qualitative studies may include a more heterogeneous sample that may complement the current vision gathered and generate new insights for the continuous implementation and sustainability of the network. One example of this effort is the preparation of face‐to‐face focus groups with older patients with other respiratory conditions than asthma in the interior of Portugal during 2023. Although central, we recognise that the patient and public perspective is not enough for cocreating a framework for supporting a PPI initiative. Therefore, it would also be interesting to assess the healthcare professionals' and researchers' views in future studies.

## 5 CONCLUSIONS

Patients, carers and citizens acknowledged the relevance of the implementation of a collaborative network and demonstrated interest in active participation in every stage of the health research cycle. The generated knowledge, together with known frameworks for supporting and evaluating PPI in research, will ground the implementation of a network to promote PPI in respiratory research. Future research needs to be done on how to implement practically and meaningfully the network. A deeper knowledge of the barriers and facilitators identified in this study will support the sustainability of such an initiative in Portugal.

## AUTHOR CONTRIBUTIONS

Paula Matos, Daniela Figueiredo, Ana L. Neves, Emília D. da Costa, Cláudia C. Loureiro, José L. Boechat, António B. Reis, Pedro Simões, Luís Taborda‐Barata, João A. Fonseca, Ana Sá‐Sousa and Cristina Jácome conceived the study design. Daniela Figueiredo, Ana L. Neves, Ana Sá‐Sousa and Cristina Jácome contributed to data collection. Margarida Areia, Liliana P. Dias, Paula Matos, Daniela Figueiredo, Ana L. Neves, Ana Sá‐Sousa and Cristina Jácome contributed to data analysis and reporting. Margarida Areia and Liliana P. Dias drafted the manuscript. All authors have read and approved the final manuscript.

## CONFLICT OF INTEREST STATEMENT

The authors declare no conflict of interest.

## ETHICS STATEMENT

The study was conducted in accordance with the Declaration of Helsinki and received approval from the Ethics Committee of the Faculty of Medicine of the University of Porto (28/CEFMUP/2021). Each participant gave their electronic informed consent previously to the focus group meetings.

## Supporting information

Supporting information.

## Data Availability

The data that support the findings of this study are available from the corresponding author upon reasonable request.
