# Supplementary material for: Public involvement in chronic respiratory diseases research: A qualitative study of patients', carers' and citizens' perspectives
Source: Health Expect. 2023 Dec 25;27(1):e13917. doi: 10.1111/hex.13917 (PMC10768873; doi:10.1111/hex.13917)
Supplement: Supplementary file 1 — Supporting information. [file HEX-27-e13917-s001.docx]

# Appendix 1

1. Have you been involved in a research project as patients/carers/citizens?

- 1. What was your role?

2.What is your opinion about the importance of the involvement of patients/carers/citizens in health research for the resolution of their experienced problems?

- 1. Why? What does it mean for you?
  2. What are the decisive factors for their involvement in research?

3.What do you think about the creation of a network of patients/carers/citizens to enable their involvement in research projects?

- 1. What would be the main objectives for that network?
  2. What are the deciding factors for the successful implementation of this idea?
  3. Who should be integrated in this network?

4.What is your opinion about enrolling in this kind of network?

- 1. What could be your role in the construction and coordination if you were involved?
  2. How do you see your involvement in the different research phases?
  3. What should this network offer to get people interested in joining?
  4. What would be the main barriers for the patients/carers/citizens involvement?
  5. What methods/strategies should be implemented to ensure that the network can represent the full spectrum of patients/carers/citizens, including hard to reach and under-represented groups?
